# Applications and indications of point-of-care ultrasound in emergency department encounters involving palliative care patients

**DOI:** 10.3389/fmed.2025.1719679

**Published:** 2025-11-20

**Authors:** Kamonwon Ienghong, Korakot Apiratwarakul, Sukanya Khemtong, Lap Woon Cheung

**Affiliations:** 1 Department of Emergency Medicine, Faculty of Medicine, Khon Kaen University, Khon Kaen, Thailand; 2 Department of Emergency Medicine, Li Ka Shing Faculty of Medicine, The University of Hong Kong, Pokfulam, Hong Kong

**Keywords:** palliative supportive care, ultrasonography, emergency service, diagnostic techniques and procedures, decision making

## Abstract

**Background:**

Point-of-care ultrasound (POCUS) is a rapid bedside imaging technique that facilitates symptom-focused, targeted care for palliative patients presenting to the emergency department (ED). This study outlines the indications, ultrasound-detected diagnoses, and procedural applications of POCUS in palliative care patients at a tertiary ED in Thailand.

**Methods:**

A retrospective analysis of adult emergency department encounters at Srinagarind Hospital was conducted from January 2020 to December 2024. Patients aged 18 years or older, classified under ICD-10 Z51.5 (palliative care), with documented POCUS examinations in the electronic medical record were included. Demographic data, presenting complaints, triage levels, the purpose of POCUS (diagnostic, procedural guidance, or both), and POCUS findings as recorded by the performing emergency medicine team were collected. Descriptive statistics were used to summarize the findings.

**Results:**

Of 875 emergency department patients classified as palliative, 434 (49.6%) had documented point-of-care ultrasound (POCUS) and were included in the study. The median age was 71 years (range 64–97). Of these, 59.7% were male, and 74.9% had oncological diagnoses. High-acuity triage levels (1–2) accounted for 41.9% of cases. POCUS was primarily used for diagnostic assessment in 74.9% of cases and for procedural support in 42.2% of patients. The most frequently identified conditions were ascites (42.6%) and pleural effusion (11.1%). The most common POCUS-guided procedures were abdominal paracentesis (34.8%) and thoracentesis (7.4%).

**Conclusion:**

POCUS frequently addressed symptom-directed concerns in palliative patients in the emergency department, commonly identifying fluid-related causes of distress and enabling bedside, symptom-relieving procedures. With appropriate training, governance, and prospective evaluation of patient-centered outcomes, POCUS has the potential to enhance acute supportive care for palliative patients across Thailand.

## 1 Introduction

Palliative care is a multidisciplinary approach aimed at improving the quality of life for patients and families facing life-threatening illnesses by preventing and alleviating suffering through early identification, assessment, and treatment of pain and other physical, psychosocial, and spiritual issues (1–3). Palliative care patients often present to the emergency department (ED) with complex, symptom-oriented concerns such as dyspnea, pain, altered mental status, and fluid retention. These situations frequently require rapid bedside evaluations to distinguish reversible conditions from expected disease progression and to align interventions with the patient’s care goals. Traditional diagnostic methods can be time-consuming, require patient transport, and may lead to invasive testing that conflicts with palliative care objectives. There is a growing need for effective tools that support prompt, bedside clinical decision-making while minimizing the burden on patients and their families (4–8).

Thailand faces a growing demand for palliative care driven by an aging population and an increasing prevalence of chronic non-communicable diseases, particularly cancer. In recent decades, palliative services have expanded from tertiary hospitals to district hospitals and community-based home care models, reflecting efforts to integrate symptom-focused care into the public health system and achieve universal health coverage. Cultural factors, including strong family involvement in decision-making and Buddhist views on suffering and mortality, shape care preferences and communication practices. Despite progress, disparities remain in workforce capacity, formal training in palliative medicine, equitable access between rural and urban areas, and the reliable availability of essential medications and interventions. These limitations contribute to frequent ED visits for symptom management and urgent decision-making, underscoring the need for practical bedside tools and care frameworks that support goal-concordant, low-burden care for palliative patients in Thailand (9–11).

Point-of-care ultrasound (POCUS) offers immediate, non-invasive imaging at the bedside and has demonstrated effectiveness in emergency settings for diagnosis, procedural guidance, and monitoring. Its portability, repeatability, and ability to directly address symptom-specific concerns make it particularly valuable for symptom-directed care in critically ill populations (12–14). However, most existing literature on POCUS focuses on general or critical care populations, with limited data specifically addressing its use in palliative care (15–17). There remains a gap in the comprehensive characterization of indications, targeted anatomical regions, and clinical contexts of POCUS application in this patient group.

This study highlights the applications and indications of POCUS during emergency department encounters involving palliative care patients. Our objective is to quantify utilization patterns, identify common diagnostic targets and procedural applications, and characterize clinical scenarios in which bedside ultrasound influenced care decisions. The findings aim to clarify current practices to guide training priorities, inform guideline development, and support future research on integrating bedside imaging into goal-concordant emergency care for palliative patients.

## 2 Materials and methods

### 2.1 Study design and setting

This retrospective study was conducted in the emergency department of Srinagarind Hospital, Faculty of Medicine, Khon Kaen University, Thailand, using electronic medical records from January 2020 to December 2024. The primary objective was to describe the applications and indications of POCUS use in palliative care patients in the emergency department.

Srinagarind Hospital is a university-affiliated medical center located in Khon Kaen, Thailand, and serves as a teaching hospital for the Faculty of Medicine at Khon Kaen University. The hospital manages approximately 60,000–70,000 emergency department visits annually.

### 2.2 Participants

The study included all patients admitted to the ED from 1 January 2020 to 31 December 2024. Eligible encounters comprised patients aged ≥ 18 years classified with ICD-10 Z51.5 (palliative care) in the ED electronic medical record, accompanied by documentation of at least one clinician-conducted POCUS examination during the ED visit. We employed ICD-10 Z51.5 to identify presumptive palliative encounters, as it is the standard institutional code for palliative care. Exclusion criteria encounters lacking a documented POCUS report or instances where POCUS documentation was unavailable for examination.

### 2.3 Data gathering

Patient demographics (age, sex), primary diagnosis category (oncologic vs. non-oncologic), presenting complaint, triage level [according to emergency severity index, ranging from level 1 (life-threatening condition) to level 5 (non-urgent condition)], vital signs at presentation, documented purpose of POCUS (diagnostic, procedural guidance, or both), POCUS-reported findings (e.g., ascites, pleural effusion, consolidation, pericardial effusion, reduced ejection fraction), and any ultrasound-guided procedures performed in the ED (e.g., paracentesis, thoracentesis, nerve blocks) were extracted from electronic medical records using the Health Object Program^®^, an authorized electronic medical records system. Each patient was assigned an anonymous identifier.

In this study, POCUS was defined as any ultrasound examination performed by a clinician at the bedside and documented in the medical record as conducted in the emergency department for a diagnostic or procedural purpose. All ultrasound examinations were performed using the Mindray M9 ultrasonography system (Mindray, Shenzhen, China).

POCUS examinations were performed by attending physicians and emergency medicine residents. Resident training in the department includes a foundational ultrasound curriculum; those involved in POCUS had completed at least 1 month of ultrasound training, comprising both didactic sessions and supervised scanning. Attending physicians had more than 5 years of experience with POCUS. The approach to performing POCUS was at the discretion of the individual clinician. During the study period, there was no centralized image archive or mandatory blinded review. POCUS findings were based on the documentation provided by the interpreting clinician.

Two independent investigators compiled and organized the data into a research database, reviewing and removing any duplicate entries. A subsequent round of data entry was conducted. In cases of discrepancies, a senior emergency physician was consulted to verify and finalize the data.

### 2.4 Statistical analysis

The sample size was determined using the following formula (18). The estimated value for P was based on data from a previously published study (19), leading to the conclusion that a minimum sample size of 340 participants was required. As this was a retrospective study, we included all eligible ED encounters with documented POCUS between January 2020 and December 2024, yielding 434 patients for analysis (exceeding the required sample size), which increases the precision of our estimates. Statistical analysis was performed using SPSS version 27.0 for Windows (SPSS Inc., Chicago, IL, United States), under license from Khon Kaen University. Unless otherwise specified, continuous variables are reported as mean and standard deviation (SD), while categorical variables are presented as number (n) or frequency (percent).

## 3 Results

The study was conducted over a 5-year period and included 875 emergency department patients diagnosed as palliative according to ICD-10 criteria. A total of 434 patients (49.6%) who underwent POCUS examinations and had complete ultrasound result records were evaluated. Among the 434 patients studied, 121 (27.88%) experienced two or more ED visits during the observation period. Of the individuals with multiple visits, 64 (52.89%) underwent multiple POCUS examinations. Out of 434 patients, 259 (59.67%) were male. The median age was 71 years (range: 64–97 years). Triage data showed that 182 patients (41.9%) were classified in higher acuity levels (1–2). The majority of underlying diagnoses were oncologic, with 325 patients (74.9%) diagnosed with cancer. Among the 434 patients, 61.75% were discharged from the ED (Table 1).

**TABLE 1:** Variables of palliative care patients who receiving POCUS examination at ED (*N* = 434).

Variables	*n* (%)
Male	259 (59.67)
Median age	71 (64–97)
**Triage level**	
1 and 2	182 (41.93)
3, 4, and 5	252 (58.06)
**Sign and symptom presented at ED**	
Tachypnea/dyspnea	203 (46.77)
Abdominal pain/discomfort	195 (44.93)
Hypotension	32 (7.37)
Peripheral edema	4 (0.92)
**Diagnosis**	
Cancer	325 (74.88)
Non-cancer	109 (25.12)
**Disposition of patients**	
Admitted	108 (24.88)
Discharged	268 (61.75)
Transferred	52 (11.98)
Died in ED	6 (1.39)

POCUS was primarily used for diagnostic purposes in 325 patients (74.9%). The most common ultrasound-detected findings were ascites in 185 patients (42.6%) and pleural effusion in 48 (11.1%). POCUS-guided procedures were performed in a subset of patients, including abdominal paracentesis in 151 cases (34.8%) and thoracentesis in 32 cases (7.4%) (Table 2).

**TABLE 2:** Characteristics of POCUS used in palliative care patients at ED.

Application	*n* (%)
For diagnosis	325 (74.88)
For perform procedure	183 (42.16)
**Diagnosis made by POCUS**	
Ascites	185 (42.63)
Pleural effusion	48 (11.05)
Pericardial effusion	4 (0.92)
Congestive heart failure	22 (5.06)
Pneumonia	35 (8.06)
Pneumothorax	3 (0.69)
Pulmonary embolism	10 (2.30)
Deep venous thrombosis	6 (1.38)
Bowel obstruction	5 (1.15)
Urinary obstruction	7 (1.61)
No diagnosis	109 (25.11)
**Procedure made by POCUS**	
Abdominal paracentesis	151 (34.79)
Thoracocentesis	32 (7.37)
Nerve block	0 (0)
No intervention	251 (57.83)

Among oncological patients, 289 (88.92%) underwent diagnostic POCUS and 152 (46.76%) underwent interventional POCUS. Among non-oncological patients, 36 (33.02%) underwent diagnostic POCUS and 31 (28.44%) underwent interventional POCUS.

## 4 Discussion

This retrospective study of 434 palliative care patients who underwent POCUS in a Thai tertiary emergency department (ED) found that POCUS was primarily used for diagnostic purposes (74.9%) and frequently identified clinically actionable conditions, notably ascites (42.6%) and pleural effusion (11.1%). POCUS also facilitated bedside procedures, with abdominal paracentesis performed in 34.8% and thoracentesis in 7.4% of cases (19–21). The observed rates of ultrasound-guided paracentesis and thoracentesis reflect real-world utilization of POCUS in an ED that integrates palliative principles into acute care, highlighting its procedural role in symptom relief. These findings align with previous research demonstrating the utility of POCUS in outpatient settings for diagnosing ascites (64.3%) and pleural effusion (16.7%). The ability to detect significant fluid accumulation via POCUS directly enables timely interventions—such as paracentesis or thoracentesis—that can reduce dyspnea, alleviate pain, and enhance overall comfort, supporting goal-concordant care (19–22). Furthermore, the notable proportion of patients triaged at high acuity (41.9% at levels 1–2) underscores the critical need for rapid, bedside clinical decision-making in this population, a need that POCUS effectively addresses.

While most POCUS literature (12, 13, 17, 23–25) focuses on general emergency or critical care populations, our study contributes to the growing recognition that POCUS aligns well with the goals of palliative care by enabling rapid, bedside evaluation of reversible or treatable sources of distress. This approach reduces the need for prolonged or burdensome transfers and investigations. Additionally, POCUS may lessen reliance on radiology resources, limit intra-hospital patient transport, and reduce time to intervention—advantages that are especially meaningful for fragile palliative patients, for whom minimizing procedural burden is a critical aspect of care.

Thailand is facing increasing demands for palliative care services due to an aging population and the growing prevalence of non-communicable diseases. Although palliative care capacity has expanded from tertiary centers to district and community settings, gaps remain in workforce training, resource distribution, and access to procedures and essential medications (9–11). Our study identified no occurrences of pain management utilizing POCUS, such as ultrasound-guided nerve blocks, which are currently a novel application for pain management globally (26–31). Expanding the use of POCUS in ED and district hospitals could reduce unnecessary transfers to tertiary centers, enable earlier symptom relief in community settings, and support local clinicians in delivering palliative care in line with Thailand’s universal health coverage goals. Collaboration among Thai emergency medicine, palliative care, and radiology societies could help standardize practices, support credentialing, and ensure the safe and effective implementation of POCUS services in community-based care.

This study has several important limitations. (1) Case identification relied on ICD-10 coding (Z51.5), which may not accurately capture the full palliative care population. (2) Only encounters with documented POCUS were included, introducing selection bias toward better-documented or more intervention-focused cases. (3) POCUS findings depended on the interpreting clinician’s documentation, with inconsistent image archiving and limited confirmatory imaging, introducing potential operator-dependent variability and misclassification. (4) The study did not include standardized patient-centered outcomes (e.g., symptom scores, quality-of-life measures), time-to-intervention data, or systematic monitoring for complications, limiting the ability to assess clinical effectiveness or safety (5) The single-center design restricts generalizability, particularly to settings with different training levels, resources, or equipment availability. (6) This study did not disclose the overall utilization of POCUS in the ED and did not compare the general emergency department population with palliative care patients. (7) A significant segment of our research timeframe overlapped with the COVID-19 pandemic. The pandemic may have impacted emergency department utilization, patient care-seeking behavior, case mix, and clinician practice patterns, potentially influencing the use of POCUS.

## 5 Conclusion

Our study demonstrated that POCUS was frequently used as a rapid diagnostic tool, often identifying treatable causes of distress—particularly ascites and pleural effusion—and enabling bedside, symptom-relieving interventions. The findings suggest that POCUS is an effective, low-burden modality that supports goal-concordant, bedside decision-making in high-acuity palliative cases, potentially reducing the need for patient transfers and resource-intensive imaging. Broader implementation in Thai emergency departments could enhance the capacity for timely symptom relief; however, safe expansion requires standardized training, image review and quality assurance mechanisms, and institutional protocols for ultrasound-guided procedures.

## Data Availability

The raw data supporting the conclusions of this article will be made available by the authors, without undue reservation.
